# Epileptic seizure forecasting with wearable‐based nocturnal sleep features

**DOI:** 10.1002/epi4.13008

**Published:** 2024-07-09

**Authors:** Tian Yue Ding, Laura Gagliano, Amirhossein Jahani, Denahin H. Toffa, Dang K. Nguyen, Elie Bou Assi

**Affiliations:** ^1^ Centre de Recherche du Centre hospitalier de l'Université de Montréal (CRCHUM) Montréal Québec Canada; ^2^ Department of Neuroscience Université de Montréal Montréal Québec Canada

**Keywords:** epilepsy, forecasting, machine learning, sleep analysis, wearable

## Abstract

**Objective:**

Non‐invasive biomarkers have recently shown promise for seizure forecasting in people with epilepsy. In this work, we developed a seizure‐day forecasting algorithm based on nocturnal sleep features acquired using a smart shirt.

**Methods:**

Seventy‐eight individuals with epilepsy admitted to the Centre hospitalier de l'Université de Montréal epilepsy monitoring unit wore the Hexoskin biometric smart shirt during their stay. The shirt continuously measures electrocardiography, respiratory, and accelerometry activity. Ten sleep features, including sleep efficiency, sleep latency, sleep duration, time spent in non‐rapid eye movement sleep (NREM) and rapid eye movement sleep (REM), wakefulness after sleep onset, average heart and breathing rates, high‐frequency heart rate variability, and the number of position changes, were automatically computed using the Hexoskin sleep algorithm. Each night's features were then normalized using a reference night for each patient. A support vector machine classifier was trained for pseudo‐prospective seizure‐day forecasting, with forecasting horizons of 16‐ and 24‐h to include both diurnal and nocturnal seizures (24‐h) or diurnal seizures only (16‐h). The algorithm's performance was assessed using a nested leave‐one‐patient‐out cross‐validation approach.

**Results:**

Improvement over chance (IoC) performances were achieved for 48.7% and 40% of patients with the 16‐ and 24‐h forecasting horizons, respectively. For patients with IoC performances, the proposed algorithm reached mean IoC, sensitivity and time in warning of 34.3%, 86.0%, and 51.7%, respectively for the 16‐h horizon, and 34.2%, 64.4% and 30.2%, respectively, for the 24‐h horizon.

**Significance:**

Smart shirt‐based nocturnal sleep analysis holds promise as a non‐invasive approach for seizure‐day forecasting in a subset of people with epilepsy. Further investigations, particularly in a residential setting with long‐term recordings, could pave the way for the development of innovative and practical seizure forecasting devices.

**Plain Language Summary:**

Seizure forecasting with wearable devices may improve the quality of life of people living with epilepsy who experience unpredictable, recurrent seizures. In this study, we have developed a seizure forecasting algorithm using sleep characteristics obtained from a smart shirt worn at night by a large number of hospitalized patients with epilepsy (78). A daily seizure forecast was generated following each night using machine learning methods. Our results show that around half of people with epilepsy may benefit from such an approach.

**Key Points**

- Investigate seizure‐day forecasting based on sleep features assessed using a smart shirt and machine learning.
- A total of 78 people with epilepsy wore the Hexoskin smart shirt during their stay at the epilepsy monitoring unit.
- Sleep features were analyzed and a SVM classifier was trained to forecast seizure days.
- Improvement over chance was achieved in 48.8% and 40% of patients for 16‐ and 24‐h seizure‐day forecasting, respectively.
- Smart shirt‐based sleep analysis shows promise for non‐invasive seizure forecasting, leading to innovative seizure advisory devices.

## 1 INTRODUCTION

Epilepsy is characterized by the occurrence of spontaneous and recurrent seizures and affects approximately 60 million individuals worldwide.^1^ Despite the availability of various antiseizure medications, one‐third of people with epilepsy (PWE) do not achieve adequate seizure control.^2^, ^3^ Uncontrolled seizures negatively impact the quality of life of PWE by increasing the risk of injury and heightening stress levels.^4^ These challenges have motivated extensive research in seizure forecasting which could significantly improve the lives of PWE and their caregivers.^2^

Over the past few decades, previous studies have shown promising evidence for the development of automated seizure forecasting algorithms which could potentially be implemented into closed‐loop intervention or advisory devices.^5^ Traditional seizure prediction approaches predominantly rely on invasive intracranial electroencephalography (EEG) recordings which may only be suitable for PWE with well‐localized focal epilepsy.^6^, ^7^, ^8^, ^9^, ^10^ Unfortunately, there are practical challenges limiting the translation of EEG‐based seizure prediction algorithms to clinical use such as the invasive nature and computational complexity of intracranial EEG.^11^ While important milestones have been reached with intracranial EEG recordings such as the discovery of cyclical patterns in epileptic activity^12^, ^13^ and network involvement in seizures,^14^ the international seizure forecasting community has recently shifted paradigm by exploring whether the information provided by noninvasive multimodal wearable devices could be used for seizure forecasting.^15^, ^16^, ^17^, ^18^

Indeed, wearables have the potential to benefit a larger and more representative population of PWE.^18^ In addition, wearables are more practical and preferred by PWE, as compared to intracranial EEG‐based implantable devices.^16^ Subsequently, several studies have used wearables to detect epileptic seizures, with promising performances for the detection of generalized and focal to bilateral tonic–clonic seizures.^18^ While only a few studies have explored the use of wearables within the context of epileptic seizure forecasting, promising results have been demonstrated using wrist‐worn devices in both the epilepsy monitoring unit (EMU) and residential settings.^19^, ^20^, ^21^, ^22^ Conducting new trials with different wearable devices, biomarkers, recording durations, and forecasting horizons may yield similar outcomes while providing PWE with a wider range of seizure forecasting tools suitable to each individual's needs.

Interestingly, in addition to their capability of recording physiological signals, such as electrocardiography and respiration, novel wearables can quantitatively assess sleep quality, which may be leveraged to forecast seizures non‐invasively.^23^ The intricate bidirectional relationship between sleep and seizures in PWE has been explored over the past three decades. Sleep duration has been found to correlate with seizure frequency, with sleep deprivation known to provoke seizures and often utilized for clinical purposes in the EMU.^2^, ^24^ On the other hand, antiseizure medications and other epilepsy treatments, such as deep brain stimulation of the anterior nucleus thalamus, can disrupt normal sleep architecture.^25^, ^26^ Payne et al.^27^ explored the use of sleep metrics as a predictive prior probability in a Bayesian model for seizure forecasting through a post hoc residential intracranial EEG study, achieving above‐chance performance in five of their eight PWE. In line with these findings, our group recently observed a decrease in sleep efficiency preceding epileptic seizures in a cohort of 47 PWE in the EMU using the Hexoskin smart shirt (Carré Technologies Inc.), showing its potential for seizure risk forecasting.^28^ Considering the complex interaction between sleep architecture and seizure frequency, machine learning algorithms hold promise in disentangling the underlying components.^15^

This study aims to assess the feasibility of combining smart shirt‐based nocturnal sleep features and machine learning for seizure‐day forecasting, with 16‐ and 24‐h horizons. We developed a seizure‐day forecasting algorithm based on nocturnal sleep data collected from a large cohort of PWE in the EMU, wearing a multimodal smart shirt.

## 2 MATERIALS AND METHODS

### 2.1 Patient recruitment

Patients hospitalized in the Centre hospitalier de l'Université de Montréal (CHUM) EMU between April 2019 and July 2022 were recruited for the study. The only inclusion criterion was patients with a confirmed diagnosis of epilepsy. Patients who experienced seizures every day or night were excluded from the analysis. For included patients, the following nights were removed, which are detailed in later sections of the manuscript: first night at the EMU, full or partial sleep deprivation, significant sleep during the preceding day, video‐EEG recording loss and smart shirt data processing failure. Patients with less than two valid nights were also excluded from the analysis. The study received approval from the Institutional Ethics Research Board (18.091), and written informed consent was obtained from each participant before their participation.

### 2.2 Signal acquisition

#### 2.2.1 Multimodal recordings

**FIGURE 1:**
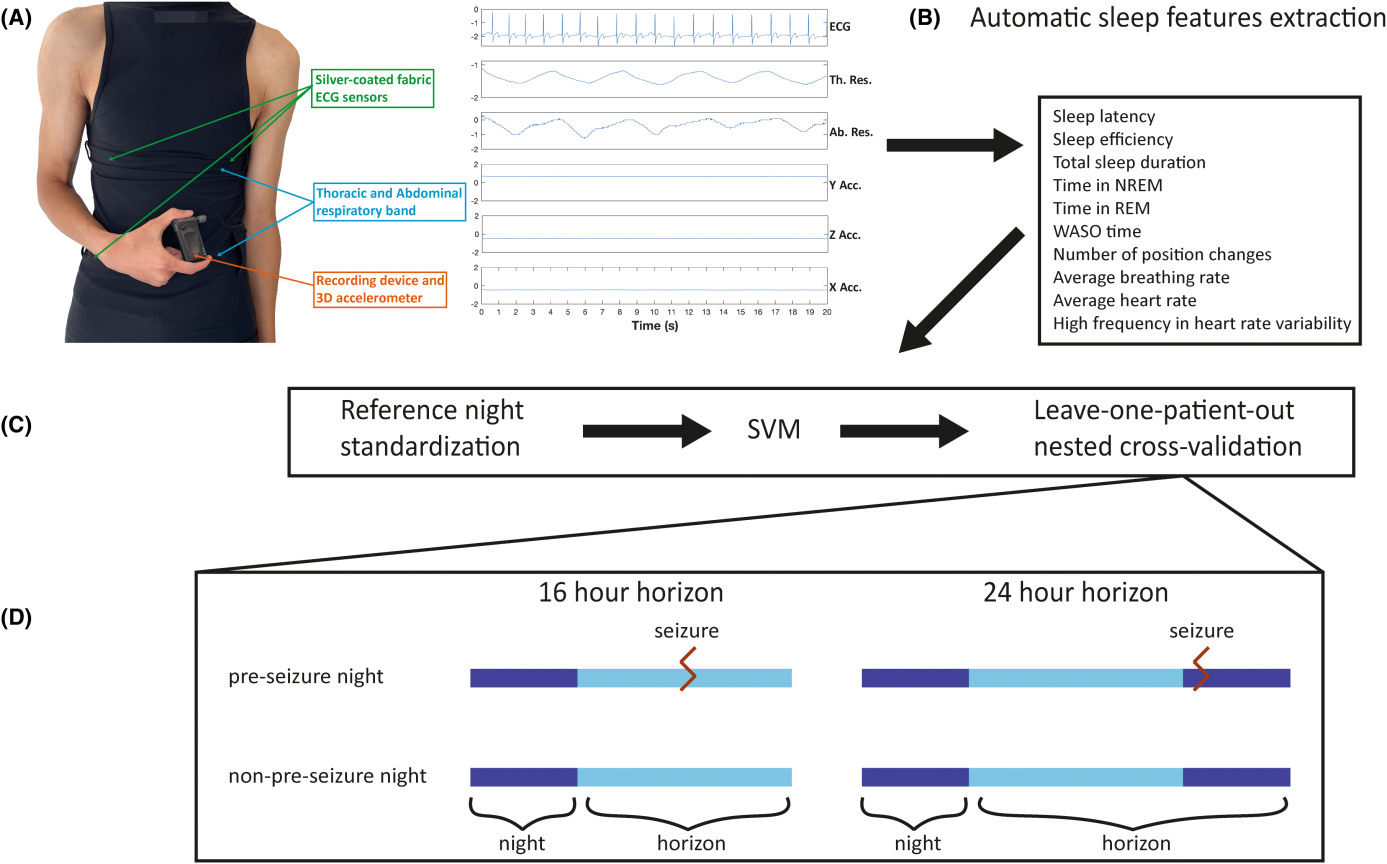
Machine‐learning model development strategy. (A) Hexoskin shirt equipped with an ECG sensor (256 Hz), respiratory bands (128 Hz each), and a 3‐axis accelerometer (64 Hz) and example raw signal segment. Ab. Res., Abdominal respiration; ECG, Electrocardiogram; Th. Res., Thoracic respiration; X Acc., X‐axis accelerometer; Y Acc., Y‐axis accelerometer; Z Acc., Z‐axis accelerometer. (B) Automatic sleep features extraction from Hexoskin's sleep algorithm. NREM, Non‐rapid eye movement sleep; REM, Rapid eye movement sleep. (C) Forecasting model development and evaluation. SVM, Support vector machine. (D) Forecasting of pre‐seizure and non‐pre‐seizure nights for both 16‐ and 24‐h horizons.

Patients wore the Hexoskin smart shirt (Carré Technologies Inc.) during the entire participation period, except when they showered or underwent imaging studies. The Hexoskin sizing guide for men and women was used to ensure the proper fitting of the shirt. The smart shirt recorded continuous physiological signals, including a single‐lead electrocardiogram (ECG) at a sampling rate of 256 Hz, two respiratory inductance plethysmography bands (thoracic and abdominal) sampled at 128 Hz each, and a three‐axis accelerometer sampled at 64 Hz. Signal quality was visually inspected daily by the research team, who adjusted the shirt fit as needed according to the Hexoskin sizing chart. None of the nights in our present study were excluded based on this inspection process. Artifact correction was automatically applied by the Hexoskin sleep analysis algorithm before calculating sleep features. Unfortunately, we do not have direct access to the specific artifact rejection process used by the algorithm. Thus, it was not possible to investigate how often artifacts occurred during the analyzed nights. Nevertheless, the analysis was conducted on nocturnal data only, which typically contain fewer artifacts than diurnal data. In addition, we ensured the proper fitting of the shirt, according to the Hexoskin sizing chart and we visually inspected recorded signals' quality daily. None of the nights in our present study were excluded based on this inspection process. Recorded data was stored in a telemetry device, which was replaced every 24 h by research personnel. Data from the telemetry device were then uploaded to the Hexoskin Connected Health Platform for further analysis (Figure 1A).

### 2.3 Data selection and classification

#### 2.3.1 Data annotation

Seizures were annotated by neurologists who were blinded to the Hexoskin data based on simultaneous video‐EEG recordings using the Nihon Kohden system. For the development of the forecasting algorithm, nights were labeled as either pre‐seizure or non‐pre‐seizure. A pre‐seizure night was defined as a night followed by at least one epileptic seizure within the forecasting horizon after waking up (Figure 1D).

#### 2.3.2 Nocturnal data selection and segmentation

Nights were excluded for each patient according to the following criteria: (1) the first night at the EMU,^29^ (2) full or partial sleep deprivation, and (3) significant sleep (>4 h) during the preceding day. Video recordings were visually analyzed to determine the lights off and lights on times, which were defined as the moments when the patient expressed an intention to sleep (e.g., by putting away their phone and lying on the bed) and when the patient no longer expressed a desire to sleep (e.g., by inclining or leaving their bed), respectively. Recordings were segmented based on the manual labeling of sleep onset (i.e., lights off) and sleep offset (i.e., lights on). Nocturnal recordings (period between sleep onset and sleep offset) were analyzed in this study.

### 2.4 Sleep feature extraction

The Hexoskin sleep algorithm automatically extracted sleep‐related features from the nocturnal data recordings. This algorithm has been previously validated and demonstrated high agreement with standard polysomnography (PSG) recordings for sleep–wake binary classification (90.8% agreement) and classification of rapid eye movement (REM) sleep, non‐rapid eye movement (NREM) sleep, and wake (77.4% agreement) in young healthy adults.^30^ A total of ten sleep‐related features were automatically calculated by the Hexoskin algorithm and used for the forecasting algorithm. These features included sleep latency, sleep efficiency, total sleep duration, time spent in NREM sleep, time spent in REM sleep, time spent awake after sleep onset (WASO), number of position changes, average breathing rate, average heart rate, and the percentage of high frequency (0.15–0.4 Hz) in heart rate variability (Figure 1B). The equations for the three calculated features are:

(1) WASO=time in wake−sleep latency

(2) $$\begin{array}{cc} \text{Total sleep duration}= & \text{time in sleep}\;\left(\mathrm{REM}+\text{NREM}\right)\\ & +\;\text{time in WASO} \end{array}$$

(3) $$\text{Sleep efficiency}=\left(1–\text{WASO}\right)/\text{Total sleep duration}$$

### 2.5 Sleep feature standardization

To account for individual variability in sleep characteristics, we calculated the relative differences of each sleep‐related feature for each night as follows:

(4) $$\text{Relative difference}=\left(x–x_{\text{reference}}\right)/x_{\text{reference}}$$

The reference night for each patient was chosen prior to algorithm development as the first night without a nocturnal seizure nor a seizure in the previous 16 h of the day, both of which negatively impact the sleep structure.^31^ The first night without a seizure was selected if such a night did not exist. Reference nights were excluded from the forecasting algorithm to avoid bias. This approach allowed us to incorporate individual differences in the forecasting algorithm.

### 2.6 Forecasting algorithm architecture

General machine learning models across patients classifying nights as either pre‐seizure or non‐pre‐seizure were developed to forecast horizons in which at least one seizure occurred. Two different forecasting horizons following wake were evaluated (16‐h and 24‐h) (Figure 1D). These horizons were selected according to literature‐reported patient preferences, as well as for practical considerations of forecasting periods including both diurnal and nocturnal seizures (24‐h) or diurnal seizures only (16‐h).^17^

#### 2.6.1 Machine learning algorithms

Regularized support vector machines (SVM) with Gaussian radial basis function kernels were used due to their robustness, ease of training, and promising performances for classification tasks.^32^ SVM hyperparameters (cost and gamma) were optimized in the inner loop of the nested cross‐validation using random search with 1000 iterations,^33^ with tuning spaces adapted from Probst et al. 2019.^34^

Analyses were performed with Python (version 3.8.10) and using the *sklearn* library (version 1.1.2).^35^

#### 2.6.2 Training, validation, and test data

A nested leave‐one‐patient‐out cross‐validation approach was employed for algorithm development for both forecasting horizons. In this approach, the inner loop consisted of all but one PWE for training and validation, where a leave‐one‐patient‐out cross‐validation was performed, while the outer loop included the single remaining patient for testing (Figure 1C). We ensured that the validation and test sets included patients who had both pre‐seizure and non‐pre‐seizure nights. This approach maximizes the testing set data^20^ and provides unbiased performance estimates.^36^

#### 2.6.3 Performance metrics

The forecasting performance was evaluated using the improvement over chance (IoC) metric, defined as the sensitivity (S) minus the time in warning (TiW).^20^, ^37^, ^38^ IoC was optimized in the inner cross‐validation loop as described in Section 2.6.1. Additionally, the area under the receiver operating curve (ROC‐AUC) was used as a secondary forecasting performance metric.

### 2.7 Post‐hoc analysis

#### 2.7.1 Post‐hoc analysis for IoC performance

To identify possible differences between patients with IoC performance and those without, we conducted a post‐hoc analysis between the two groups for the number of seizure days, the number of days in the study, age and sex. An appropriate statistical test was applied following the assessment of data distribution for each of the numerical variables. A Chi‐squared test was applied for the categorical variable of sex. Statistical significance was set to *p* = 0.05.

#### 2.7.2 Post‐hoc analysis for sex

We then conducted a statistical analysis to determine whether there were differences in the variables of the number of seizure days, the number of days in the study, age, and the proportion of patients with IoC performance between males and females. An appropriate statistical test was applied following the assessment of data distribution for each of the numerical variables. A Chi‐squared test was conducted for the categorical variables of the proportion of patients of each sex with IoC performance.

## 3 RESULTS

### 3.1 Demographics

**FIGURE 2:**
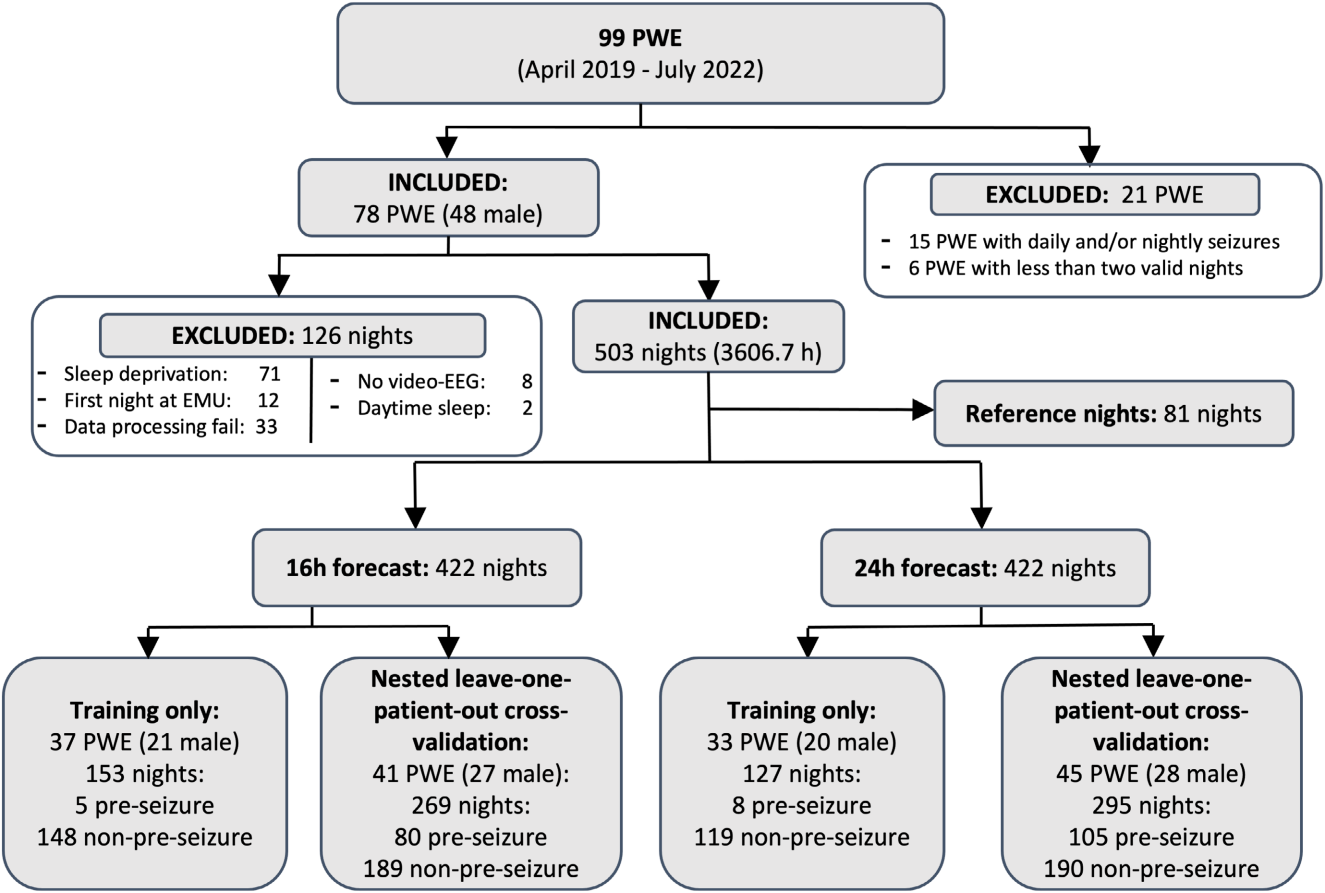
Flow diagram illustrating participant selection and group distribution for forecasting algorithm development. A total of 99 patients were recruited at our Epilepsy Monitoring Unit between April 2019 and July 2022. Of these, 78 were included and analyzed in this study. Patients with both pre‐seizure and non‐pre‐seizure nights were included in the validation folds of the leave‐one‐patient‐out nested cross‐validation folds, while patients with only non‐pre‐seizure nights were included in the training folds only. PWE, People with epilepsy; Pre‐seizure night, Night which was followed by one or more epileptic seizure in the specified forecasting horizon following wake; non‐pre‐seizure night, Night which was not followed by an epileptic seizure in the specified forecasting horizon following wake.

Ninety‐nine patients with epilepsy hospitalized in the CHUM EMU between April 2019 and July 2022 were recruited to wear the Hexoskin smart shirt. Fifteen of them were excluded for having daily seizures to avoid over‐optimistic results. Another six were excluded for having less than the minimum of two valid nights which are required for analysis, according to exclusion criteria (Section 2.1, Figure 2).

In total, 78 patients were included for the development and testing of the 16‐ and 24‐h horizon forecasting algorithms, respectively. On average, the shirt was worn for 8.1 days (range = [3, 24]). There were 48 males and 30 females (mean ± SD age: 35.15 ± 12.23 years, range = [18, 66]), with both focal (73/78) and generalized (6/78) epilepsy (one patient had both generalized and multifocal seizures). The confirmed or suspected localization of the epileptic focus in focal epilepsy patients is provided in the Table S1. The number of seizures and the total recording time per patient are also provided (Table S1).

A total of 422 nights were analyzed in both forecasting horizons following exclusion criteria (Figure 2). There were 85 pre‐seizure and 337 non‐pre‐seizure nights in the 16‐h horizon, and 113 pre‐seizure and 309 non‐pre‐seizure nights in the 24‐h horizon. Forty‐one and 45 patients with both pre‐seizure and non‐pre‐seizure nights were included in the validation and test sets for the 16‐ and 24‐h forecasting horizons, respectively (number of pre‐seizure nights/total nights: 80/269 and 105/295).

### 3.2 Forecasting performance

Seizure forecasting performances were evaluated in terms of sensitivity, TiW, IoC, and ROC‐AUC for the forecasting horizons of 16‐ and 24‐h, following the nested leave‐one‐patient‐out cross‐validation approach described in Section 2.6.

#### 3.2.1 Forecasting horizon of 16‐h

**FIGURE 3:**
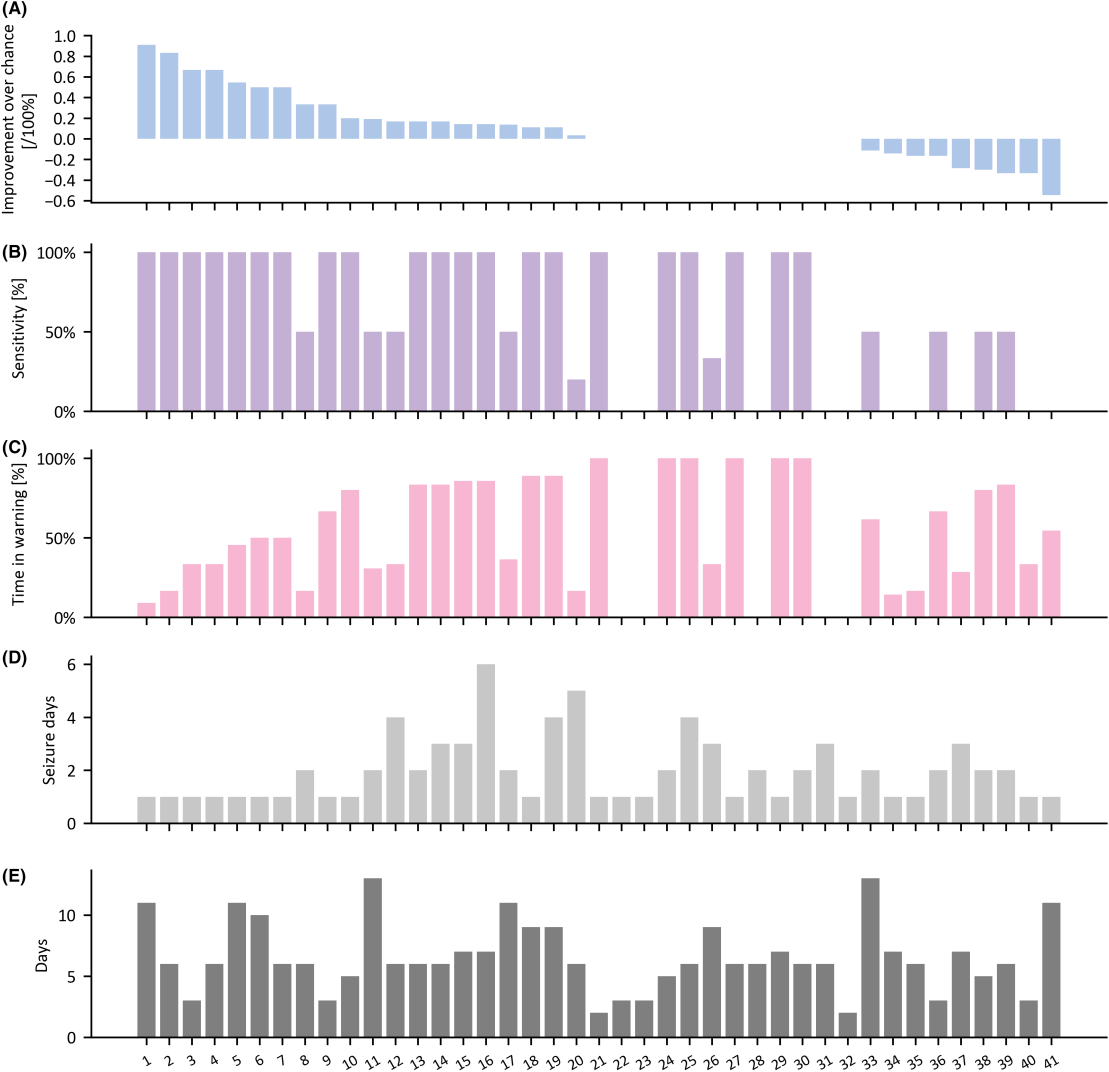
Forecasting performances for the 41 people with epilepsy of the test folds of the 16‐h forecast horizon.

Seizure forecasting achieved better than chance performance for 48.8% of patients (20 of 41 patients), for whom the mean IoC, sensitivity, and TiW were 34.3 ± 26.4%, 86.0 ± 25.6%, and 51.7 ± 28.4%, respectively (mean ± SD) (Figure 3). Including all 41 patients, after setting negative IoC values to zero in the same manner as Meisel et al.^20^ the mean IoC, sensitivity, and TiW were 16.7 ± 25.1%, 62.3 ± 42.6% and 51.4 ± 34.9%, respectively (mean ± SD). The mean ROC‐AUC were 0.707 ± 0.225 and 0.590 ± 0.289 for the 20 PWE with IoC and all 41 PWE, respectively.

#### 3.2.2 Forecasting horizon of 24‐h

**FIGURE 4:**
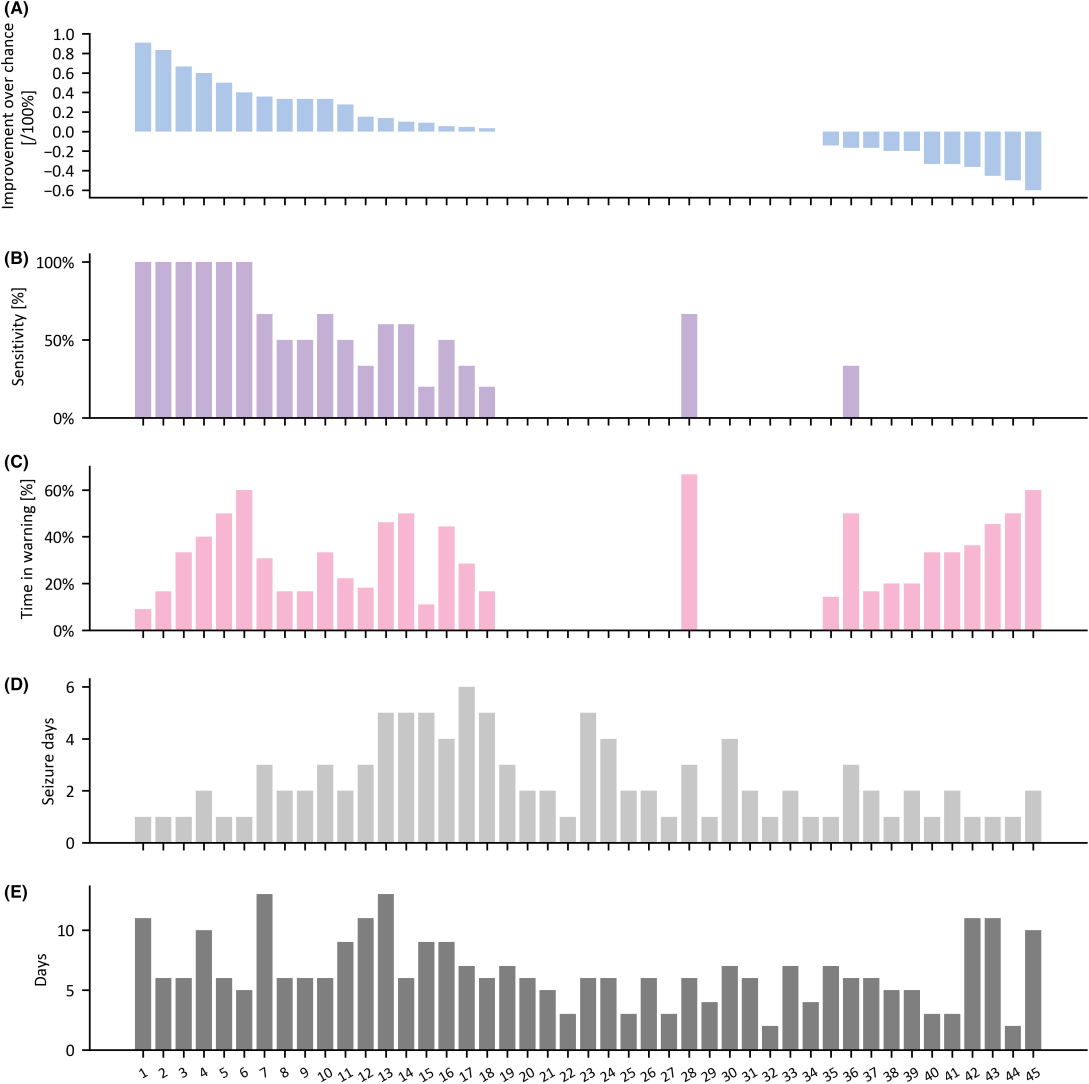
Forecasting performances for the 45 people with epilepsy of the test folds of the 24‐h forecast horizon.

Seizure forecasting achieved better than chance performance for 40.0% of patients (18 of 45 patients), for which the mean IoC, sensitivity, and TiW were 34.2 ± 26.9%, 64.4 ± 29.1%, and 30.2 ± 15.3%, respectively (mean ± SD) (Figure 4). Including all 45 patients, after setting negative IoC values to zero in the same manner as Meisel et al.,^20^ the mean IoC, sensitivity, and TiW were 13.7 ± 23.8%, 28.0 ± 36.7%, and 22.0 ± 20.6%, respectively (mean ± SD). The mean ROC‐AUC were 0.813 ± 0.170 and 0.583 ± 0.326 for the 18 PWE with IoC and all 45 PWE, respectively.

### 3.3 Post‐hoc analysis

#### 3.3.1 Post‐hoc analysis for IoC performance

In the 16‐h forecasting horizon, patients with IoC had on average more seizure days as well as more days analyzed than those without IoC (2.15 ± 1.53 > 1.76 ± 0.89 seizure days and 7.35 ± 2.74 > 5.8 ± 2.79 analyzed days), which would be consistent with the fact that machine learning algorithms performances improve with more data. However, our post‐hoc analysis using the Mann–Whitney *U*‐test did not show any significant differences between groups for these two variables (*p* > 0.05), neither in the mean age (35.95 ± 12.77 vs 34.10 ± 13.08 in the IoC and no IoC groups, respectively). The Chi‐squared test also did not show any association between sex and whether there was IoC (*p* > 0.05).

Similarly, in the forecasting 24‐h horizon, patients with IoC had on average more seizure days as well as more days analyzed than those without IoC (2.89 ± 1.71 > 1.96 ± 1.09 seizure days and 8.06 ± 2.62 > 5.56 ± 2.42 analyzed days). Interestingly, the Mann–Whitney test showed a statistically significant difference in the number of analyzed days (*p* = 0.005). The mean number of seizure days and the mean age (35.06 ± 11.01 vs 36.37 ± 14.92 for IoC and no IoC groups, respectively) were not statistically significant, neither was there any association in the Chi‐squared test for sex differences (*p* > 0.05).

#### 3.3.2 Post‐hoc analysis for sex

The algorithm achieved IoC performance in 14/27 males and 6/14 females, and 11/28 males and 7/17 females for the 16‐h and 24‐h forecasting horizons, respectively. Chi‐squared test showed no association in the proportion of each sex with improvement over chance performance (*p* > 0.05) for both forecasting horizons.

In the 16‐h horizon, males had on average less seizure days than females (1.74 ± 0.94 vs 2.36 ± 1.65) for the same number of study days (6.56 ± 2.76 vs 6.57 ± 3.08), although this difference was not statistically significant according to the Mann–Whitney *U*‐test (*p* > 0.05). In the 24‐h horizon, there was no statistical difference (*p* > 0.05) between males and females in the mean number of seizure days (2.18 ± 1.12 vs 2.59 ± 1.84) and of study days (6.79 ± 2.70 vs 6.18 ± 2.92). No differences were found in the mean age between groups for males and females in both 16‐h and 24‐h forecasting horizons (*p* > 0.05).

#### 3.3.3 Post‐hoc analysis for presumed epilepsy foci

Epilepsy types may present with different sleep and seizures patterns, which may lead to certain biases when assessing the performance of a general forecasting algorithm on a patient population with different seizure foci. Our patient cohort is mostly composed of focal epilepsies with presumed temporal lobe origin. Thus, we conducted a post‐hoc analysis comparing the algorithm performances between PWE with temporal lobe epilepsy (*n* = 47) and those with other types (frontal = 12, parietal = 1, insular = 1, multifocal = 6, generalized = 5, total *n* = 31) (Table 1). No statistically significant difference was found in IoC performances between PWE with temporal lobe epilepsy and those with other types for both forecasting horizons (*p* > 0.05 Chi‐squared test).

**TABLE 1:** Summary of presumed epilepsy types.

Presumed epilepsy type^a^	Number of PWE	Number in test set 16 h (number IoC)	Number in test set 24 h (number IoC)
Temporal	47	24 (10)	28 (11)
Frontal	12	6 (6)	6 (2)
Parietal	1	1 (0)	1 (0)
Insular	1	1 (0)	1 (0)
Multifocal	6	3 (1)	3 (1)
Generalized	5	0	0
Other^b^	6	6 (3)	6 (4)

^a^ Epilepsy types were not validated by intracranial EEG investigations.

^b^ Six PWE with two types: three frontotemporal, one temporo‐insular, one parieto‐insular, one generalized, and multifocal.

#### 3.3.4 Post‐hoc analysis for nocturnal seizures

We expect that nocturnal seizures will result in sleep disruptions increasing the time in wakefulness. While the proposed machine learning algorithm may be able to account for these changes and generate a corresponding seizure‐day forecast, we conducted a post‐hoc analysis comparing the performances of the algorithm in PWE with and without nocturnal seizures. There were 30 of 45 and 26 of 41 PWE with nocturnal seizures in the 16‐h and 24‐h forecasting horizons, respectively. No statistically significant difference was found in both horizons (*p* > 0.05 Chi‐squared test).

## 4 DISCUSSION

In this study, we developed seizure forecasting algorithms using smart shirt data collected during nocturnal periods in a relatively large group of people with epilepsy admitted to our EMU. We compared the performance of the algorithms for 16‐h and 24‐h forecasting. Approximately 40–50% of PWE displayed better‐than‐chance performance for both horizons, with slightly better results observed for the 16‐h horizon (IoC = 34.3% > 34.2% and sensitivity = 86.0% > 64.4%, respectively). Our findings align with previous studies indicating that forecasting performance tends to decrease with longer horizons.^22^, ^39^ The 16‐h horizon may be more practical for PWE who only want daytime seizures to be forecasted.

Our results show promise for the use of nocturnal sleep metrics extracted from wearable sensors, such as the Hexoskin smart shirt, placed at a distance from the central nervous system, as predictive components in seizure forecasting. Despite the relatively short duration of recordings and variability in seizure type and timing, the presented algorithm achieved better‐than‐chance classification in approximately half of patients.

Our first post‐hoc analysis showed a significant increase in the number of study days in PWE with IoC than in those without in the 24‐h horizon, which suggests that a longer number of recording days is highly important for quality algorithm development. This may be particularly crucial as the forecasting horizon increases. Our second post‐hoc analysis showed that our algorithm is unbiased in regards to sex differences. Our third post‐hoc analysis comparing the temporal epilepsies to the other patients showed that the algorithm is not biased toward the temporal epilepsies only, although our number of patients of each of the other epilepsy types is low. It may be interesting to take into account the epilepsy types in a future study, which may show better performances to assess the baseline sleep quality for each patient, as the patterns in seizure occurrence and sleep characteristics may differ between types.

The use of sleep for seizure forecasting has been explored in previous studies. Payne et al.^27^ achieved above‐chance performance in predicting seizures in 10‐minute segments of five of eight PWE using long‐term intracranial EEG data, with time since waking being the most useful sleep feature (mean ROC‐AUC = 0.630). However, comparing their study with ours is challenging due to differences in data acquisition methods and forecasting horizons. Our study achieved above‐chance performance in approximately 40–50% of PWE for fixed 16‐h or 24‐h forecasting horizons (mean ROC‐AUC for patients with IoC = 0.707 and 0.813, respectively). It is worth noting that our group previously observed decreased sleep efficiency using the same smart shirt in nights followed by at least one epileptic seizure within 24 h of awakening.^28^

IoC was selected as the primary performance metric to account for the baseline seizure chance in each PWE, following the approach of previous studies.^20^, ^39^ However, interpreting the magnitude of IoC in longer forecasting horizons can be challenging. For instance, a highly accurate algorithm may yield a relatively low IoC in a PWE with a significant proportion of seizure‐days, which is due to an extended warning time. Conversely, as we have observed in this study, the highest IoCs were achieved in PWE with only one or two seizure days.

Feature contribution analysis of our algorithms may provide some pathophysiological insight into specific sleep parameters relating to seizure risks for the following day. One common approach to understanding the importance or contribution of features in non‐linear SVM models is the feature importance method. Therefore, we have analyzed forecasting performances when a single feature is removed, thereby inferring which feature may be most contributive to the overall forecast, as done by Meisel et al.^20^ Our results are shown in File S2. Single feature contribution analysis results showed high variability between PWE due to high standard deviations in algorithm performance with the removal of each feature, which may be due to the inter‐patient variability of pre‐seizure signatures. Sleep latency appears to be an important feature in both 16 h and 24 h horizons, as can be seen by the decrease in performance with its removal (Figure S2A,B, respectively). In patients without initial IoC, feature removal did not appear to improve performances. Although we have shown significantly increased WASO in pre‐seizure nights than non‐pre‐seizure nights in a previous paper,^28^ it is highly probable that the most important features differ for each patient, and that it is the combination of the nocturnal features which allow for seizure‐day forecasting. This also supports the need for long‐term recordings for the development of patient‐specific seizure forecasting algorithms.

Our results using a smart shirt align with three recent forecasting studies that utilized smart watches or bands. However, direct comparisons are difficult due to methodological differences in data acquisition settings and forecasting horizons. Meisel et al.^20^ achieved better‐than‐chance seizure prediction for around half of the patients (30 out of 69) in an EMU setting, with mean IoC and prediction horizons of approximately 28.5% and 31.6 min, respectively. Similarly, Stirling et al.^22^ achieved better‐than‐chance performance for seizures in 7 out of 8 PWE and 4 out of 8 PWE in hourly and daily forecasts in real‐life settings, with mean ROC‐AUC values of 0.68 and 0.59, respectively. Nasseri et al.^21^ achieved better‐than‐chance performance for seizures in 5 out of 6 ambulatory patients, with a mean ROC‐AUC of 0.80 and an average alert time of 33 min. In our study, the general forecasting algorithm achieved a mean ROC‐AUC of 0.813 for PWE with IoC for the 24‐h horizon. We anticipate that patient‐specific algorithms could further improve the results.

Several studies have explored the interrelationship between sleep and seizure occurrence. Recent studies have shown that seizures in PWE may not occur randomly but on various timescales, including ultradian sleep cycles.^12^, ^13^, ^39^, ^40^ Additionally, sleep architecture, sleep–wake timing, and cortical excitability have been suggested to modulate seizure susceptibility.^41^ The complex interaction between sleep–wake architecture and seizure likelihood calls for machine‐learning approaches that can handle such multivariate relationships.^15^

The use of wearable devices, such as the smart shirt in our study, addresses the need for seizure prediction and detection using wearable technology in PWE and their caregivers.^16^, ^42^, ^43^, ^44^, ^45^, ^46^ Advantages of a device worn only during the night are the reduction of social stigma and the need for less data, although a real‐time forecast may not be possible. These devices provide opportunities for long‐term monitoring in clinical and out‐of‐hospital settings, with promising results in seizure prediction, detection, and characterization, leading to practical applications. This prediction method can potentially alleviate the anxiety associated with the unpredictable nature of seizures in PWE. Furthermore, an accurate seizure‐day forecasting algorithm may allow for chronotherapy, where antiseizure medications are adjusted according to periods with high or low risk of seizures. Innovative personalized patient follow ups may also be possible, where clinicians can monitor the seizure risk of their patients and time their medical appointments accordingly.

The development of e‐health systems, telemedicine, and mobile health applications is becoming increasingly important in healthcare.^47^ Wearable devices can contribute to continuous monitoring, diagnosis, prediction, and treatment of various medical conditions. The application of neurophysiological methods, including wearable devices, for automatic seizure detection in outpatients with epilepsy has been suggested by organizations like the International League Against Epilepsy (ILAE) and the International Federation of Clinical Neurophysiology (IFCN).^15^ However, there is still a gap between the rapid development of digital technology and conservative clinical practices.^15^, ^48^

Limitations of our study include the relatively short duration of nocturnal recordings per patient, the presence of independent seizure triggers (e.g., medication changes), and the use of a single night for assessing baseline sleep. Additionally, the general model we developed did not account for patient‐specific characteristics such as bedtimes and wake times. Furthermore, while a relatively large cohort of PWE participated in our study, patients admitted to the EMU are not necessarily representative of the general population. These limitations are inherent to the EMU setting but allowed us to confirm seizures using ground‐truth video‐EEG and develop a general forecasting algorithm using only nocturnal sleep data from a relatively large number of PWE. Future perspectives include the addition of features such as accounting for epilepsy types and combining sleep features with other known seizure triggers. Future algorithm development strategies include testing different seizure forecasting horizons for probabilistic forecasting and comparing algorithms for focal and generalized seizures. Finally, conducting long‐term studies in residential settings with better baseline assessment and medication stability would allow for patient‐specific algorithm development and feature selection.

## 5 CONCLUSION

This study showed that seizure‐day forecasting could be possible using noninvasive smart shirt‐measured nocturnal sleep data along with machine learning models in half of a relatively large cohort of patients with epilepsy at the EMU. These findings will motivate further studies in a residential setting by our group for the development and testing of long‐term patient‐specific forecasting algorithms. The exploration of different wearable monitoring modalities, the flexibility between continuous recordings or nocturnal sleep data alone, and the variable forecasting horizons may offer various options to people with epilepsy who will be able to select what is most suitable for their needs.

## FUNDING INFORMATION

This work was supported by the Canada Research Chair T2 in Epilepsy and Functional Anatomy of the Human Brain, the Canadian Institute of Health Research (CIHR), the Natural Sciences and Engineering Research Council of Canada (NSERC), the Fonds de Recherche du Québec – Nature et Technologies (FRQNT), the TransMedTech Institute (iTMT), and the Institute for Data Valorisation (IVADO). The funding sources had no involvement in this study.

## CONFLICT OF INTEREST STATEMENT

None of the authors has any conflict of interest to disclose. We confirm that we have read the Journal's position on issues involved in ethical publication and affirm that this report is consistent with those guidelines.

## ETHICS STATEMENT

The study received approval from the Institutional Ethics Research Board (18.091).

## PATIENT CONSENT STATEMENT

Written informed consent was obtained from each participant before their participation.

## Supporting information


Appendix S1.

## Data Availability

The datasets presented in this article are not readily available because sharing of raw data is conditional to approval by our Institution's Research Ethics Board. Requests to access the datasets should be directed to EB, elie.bou.assi.chum@ssss.gouv.qc.ca.
