# Supplementary material for: Epileptic seizure forecasting with wearable‐based nocturnal sleep features
Source: Epilepsia Open. 2024 Jul 9;9(5):1793–805. doi: 10.1002/epi4.13008 (PMC11450616; doi:10.1002/epi4.13008)
Supplement: Supplementary file 1 — Appendix S1. [file EPI4-9-1793-s001.zip › Ding-supportingfiles2.docx]

**Supplementary File S2**: Single feature contribution analysis

To assess the impact of each sleep-related feature, additional experiments were conducted by including all but one feature described in section 2.4 (sleep latency, sleep efficiency, total sleep duration, time spent in NREM sleep, time spent in REM sleep, time spent awake after sleep onset (WASO), number of position changes, average breathing rate, average heart rate, and the percentage of high frequency (0.15–0.4 Hz) in heart rate variability). The performance of the forecasting algorithm without that feature was evaluated using the same methodology outlined in section 2.6. This was repeated for each of the ten features.

Single feature contribution analysis results showed high variability between PWE due to high standard deviations in algorithm performance with the removal of each feature, which may be due to the inter-patient variability of pre-seizure signatures.

Sleep latency appears to be an important feature in both 16h and 24h horizons, as can be seen by the decrease in performance with its removal (Figures S2A and S2B, respectively). In the patients without initial IoC, feature removal did not improve performances.

These findings support the need for future studies with individualized features for patient-specific forecasting algorithms.


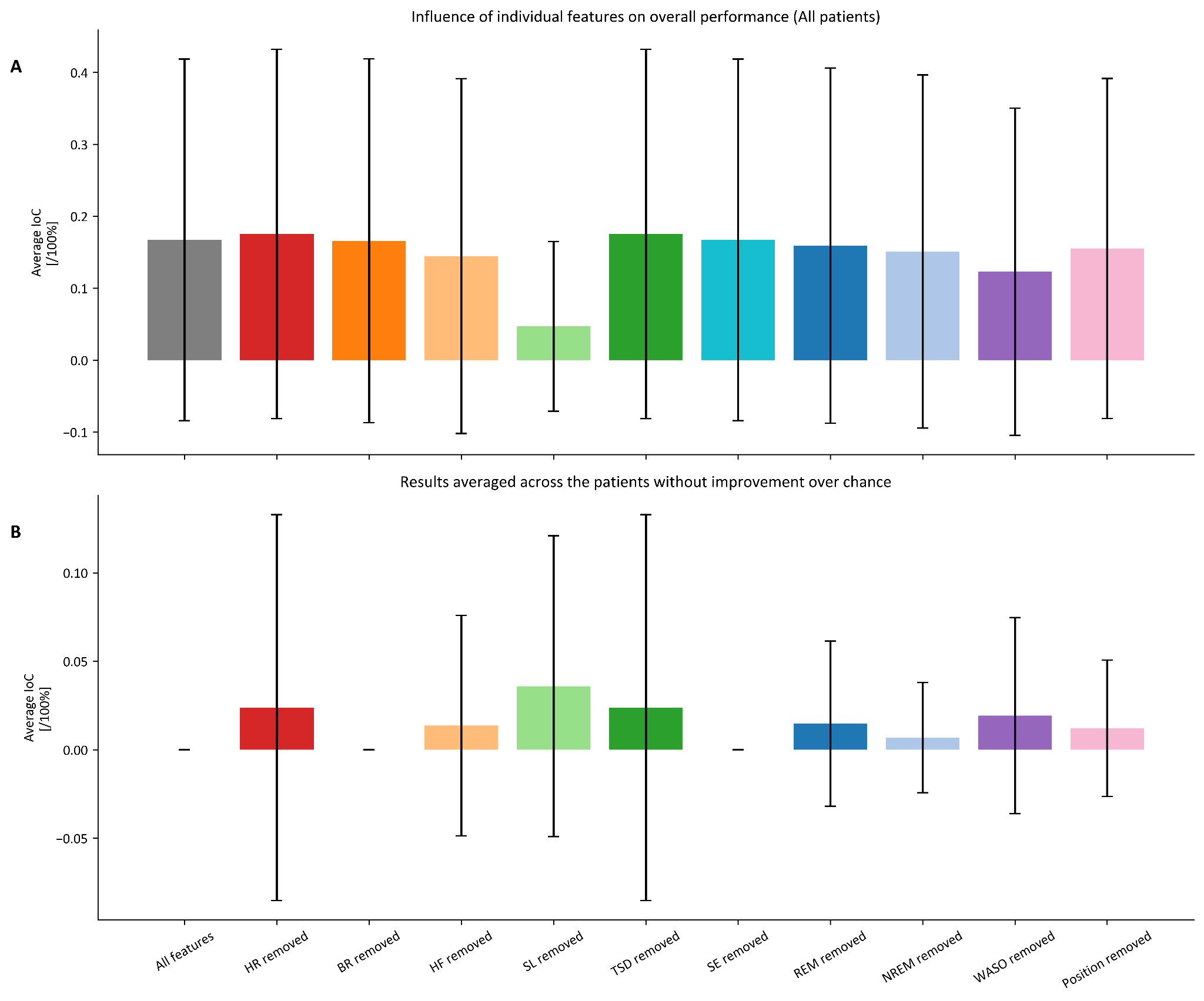


**Figure S2A**: A. Contribution of different sleep-related features to the 16h forecast horizon. B. Average results in the 21 people with epilepsy without improvement over chance. IoC: improvement over chance. HR: average heart rate; BR: average breathing rate; HF: percentage of high frequency in heart rate variability; SL: sleep latency; TSD: total sleep duration; SE: sleep efficiency; REM: rapid eye movement sleep; NREM: non-rapid eye movement sleep; WASO: time spent awake after sleep onset; Position: number of position changes.


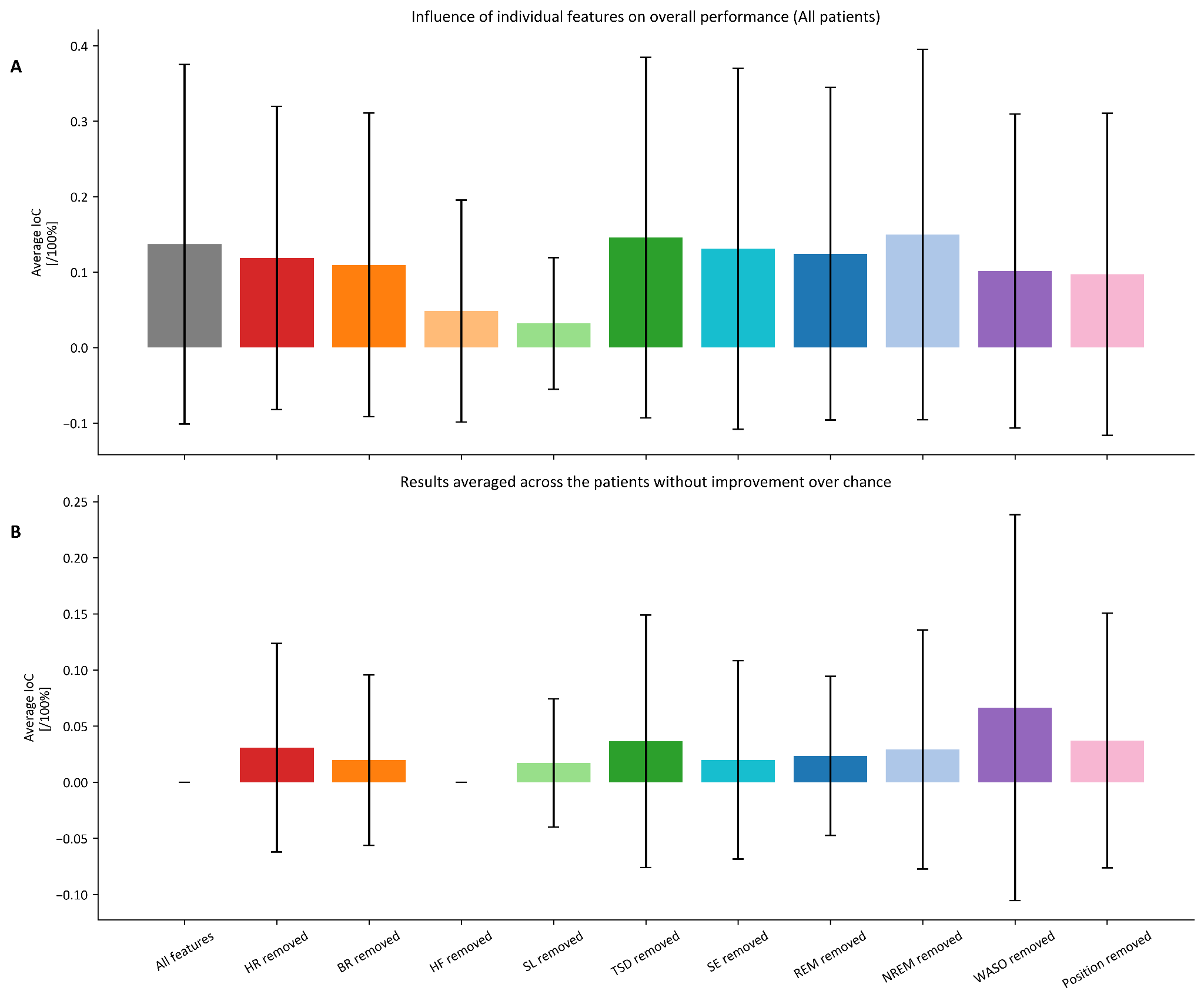


**Figure S2B**: A. Contribution of different sleep-related features to the 24h forecast horizon. B. Average results in the 27 people with epilepsy without improvement over chance. IoC: improvement over chance. HR: average heart rate; BR: average breathing rate; HF: percentage of high frequency in heart rate variability; SL: sleep latency; TSD: total sleep duration; SE: sleep efficiency; REM: rapid eye movement sleep; NREM: non-rapid eye movement sleep; WASO: time spent awake after sleep onset; Position: number of position changes.
