# Supplementary material for: Epileptic seizure forecasting with wearable‐based nocturnal sleep features
Source: Epilepsia Open. 2024 Jul 9;9(5):1793–805. doi: 10.1002/epi4.13008 (PMC11450616; doi:10.1002/epi4.13008)
Supplement: Supplementary file 1 — Appendix S1. [file EPI4-9-1793-s001.zip › Ding-supportingtables1.docx]

**Table S1:** Summary of patient clinical information

| **Patient ID** | **Sex** | **Age (yrs)** | **Presumed/confirmed epilepsy foci*** | **Main seizure type** | **Total number of seizure days (16h and 24h)** | **Total number of seizures** | **Number of analyzed nights and duration (hours)** | **IoC 16h horizon** | **IoC 24h horizon** |
| --- | --- | --- | --- | --- | --- | --- | --- | --- | --- |
| 109 | M | 47 | Bitemporal | FBTCS | 3 4 | 12 | 8 (67.4) | 0.14 | 0.00 |
| 112 | F | 32 | Right temporal | FBTCS | 3 4 | 5 | 14 (106.4) | 0.19 | 0.36 |
| 113 (1st stay) | M | 29 | Right temporal | FAS | 0 0 | 0 | 13 (87.5) | NA | NA |
| 113 (2nd stay) | M | 31 | Right temporal | FAS | 0 0 | 0 | 3 (24.9) | NA | NA |
| 113 (3rd stay) | M | 32 | Right temporal | FAS | 0 0 | 0 | 2 (16.7) | NA | NA |
| 115 | M | 49 | Right temporal | FBTCS | 1 1 | 1 | 2 (13.8) | NA | NA |
| 116 | F | 59 | Left temporal | FBTCS | 5 5 | 11 | 7 (72.5) | 0.03 | 0.03 |
| 117 | M | 38 | Generalized | GTCS | 0 0 | 0 | 5 (40.3) | NA | NA |
| 119 | M | 43 | Right frontotemporal | FIAS | 1 1 | 1 | 11 (90.8) | 0.50 | 0.60 |
| 120 | M | 58 | Left frontotemporal | FBTCS | 2 2 | 3 | 7 (61.4) | 0.33 | -0.17 |
| 123 | M | 55 | Right temporal | FIAS | 1 1 | 19 | 4 (11.5) | -0.33 | -0.33 |
| 125 | M | 22 | Generalized | GTCS | 1 1 | 0 | 2 (18.1) | NA | NA |
| 127 | M | 46 | Left temporal | FBTCS | 0 2 | 3 | 11 (97.9) | NA | -0.60 |
| 128 | F | 27 | Bitemporal | FAS, FIAS | 0 0 | 0 | 6 (43.9) | NA | NA |
| 129 | M | 28 | Left temporo-insular | FBTCS | 2 2 | 12 | 3 (28.1) | 0.00 | -0.50 |
| 130 | M | 48 | Right temporal | FBTCS (controlled), PNES | 0 0 | 0 | 5 (37.1) | NA | NA |
| 132 | F | 66 | Left temporal | FAS | 0 1 | 1 | 5 (36.9) | NA | 0.00 |
| 136 | F | 24 | Combined generalized and multifocal | GTCS, FBTCS | 1 1 | 1 | 7 (52.7) | 0.00 | 0.50 |
| 138 | F | 42 | Right temporal | FBTCS | 1 2 | 3 | 10 (84.6) | 0.11 | 0.28 |
| 140 | M | 22 | Right insular | FIAS | 2 2 | 2 | 6 (40.8) | 0.00 | 0.00 |
| 142 | F | 20 | Right frontal | FBTCS | 1 1 | 1 | 12 (85.5) | 0.55 | -0.45 |
| 145 | M | 55 | Bitemporal | FAS | 0 0 | 0 | 2 (16.0) | NA | NA |
| 147 | M | 34 | Bitemporal | FIAS, FBTCS | 2 2 | 2 | 7 (53.6) | 0.00 | 0.33 |
| 149 | F | 38 | Right temporal | FAS | 6 6 | 25 | 8 (54.6) | 0.14 | 0.05 |
| 150 | F | 53 | Left temporal | FAS | 0 0 | 0 | 4 (30.1) | NA | NA |
| 151 | M | 61 | Right temporal | FIAS | 3 4 | 15 | 8 (68.4) | -0.29 | 0.00 |
| 152 | F | 33 | Bitemporal | FBTCS | 0 0 | 0 | 13 (86.4) | NA | NA |
| 155 | M | 22 | Bitemporal | FBTCS | 2 3 | 3 | 12 (112.6) | 0.14 | 0.15 |
| 156 | M | 33 | Bitemporal | FIAS | 3 3 | 11 | 7 (50.4) | 0.00 | -0.17 |
| 157 | F | 43 | Bitemporal | FAS | 0 0 | 0 | 6 (48.4) | NA | NA |
| 158 | M | 32 | Right temporal | FAS | 0 0 | 0 | 5 (38.4) | NA | NA |
| 159 | M | 30 | Left parietal | FIAS, FBTCS | 1 2 | 3 | 4 (33.7) | 0.00 | 0.00 |
| 161 | M | 19 | Left temporal | FIAS | 5 5 | 9 | 7 (59.8) | 0.17 | 0.00 |
| 162 | M | 21 | Left frontal | FIAS, FBTCS | 0 0 | 1 | 2 (17.5) | NA | NA |
| 163 | M | 31 | Left temporal | FIAS, FBTCS | 1 2 | 2 | 7 (57.9) | 0.83 | 0.33 |
| 164 | F | 47 | Multifocal (frontotemporal ++) | FIAS | 0 0 | 0 | 5 (47.4) | NA | NA |
| 167 | F | 22 | Focal (unknown focus) | FIAS | 5 6 | 17 | 7 (47.7) | 0.00 | 0.00 |
| 168 | M | 19 | Right frontal | FBTCS | 2 2 | 3 | 7 (53.6) | 0.17 | 0.00 |
| 169 | M | 32 | Bifrontal | FAS | 1 2 | 6 | 4 (33.9) | 0.33 | 0.00 |
| 170 | F | 62 | Bitemporal | FAS | 2 2 | 18 | 4 (28.7) | -0.17 | -0.33 |
| 171 | M | 21 | Bitemporal | FIAS | 0 0 | 0 | 5 (24.0) | NA | NA |
| 172 | F | 33 | Bitemporal | FIAS | 1 1 | 6 | 4 (25.5) | 0.00 | 0.00 |
| 174 | F | 38 | Generalized | GTCS | 0 0 | 0 | 4 (28.8) | NA | NA |
| 175 | M | 27 | Frontal (unknown lateralization) | FIAS, FBTCS | 0 0 | 0 | 8 (53.4) | NA | NA |
| 176 | M | 20 | Right frontal | FIAS, FBTCS | 0 1 | 1 | 3 (24.9) | NA | NA |
| 177 | F | 42 | Right temporal | FIAS, PNES | 0 0 | 0 | 2 (16.1) | NA | NA |
| 178 | M | 23 | Right temporal | FIAS, FBTCS | 2 2 | 7 | 7 (60.7) | 0.00 | 0.00 |
| 179 (1st stay) | F | 25 | Bitemporal | FIAS, FBTCS | 3 5 | 9 | 10 (87.7) | 0.00 | 0.09 |
| 179 (2nd stay) | F | 25 | Bitemporal | FIAS, FBTCS | 0 0 | 0 | 2 (17.3) | NA | NA |
| 181 | M | 34 | Multifocal | FAS | 3 4 | 4 | 7 (41.9) | -0.33 | 0.00 |
| 182 | M | 39 | Left temporal | FIAS, FAS, FBTCS | 5 5 | 7 | 5 (34.8) | NA | NA |
| 183 | M | 32 | Left frontal | FAS, FBTCS | 1 3 | 4 | 4 (26.3) | 0.67 | NA |
| 188 | F | 48 | Generalized | GTCS, absences | 0 0 | 0 | 10 (91.0) | NA | NA |
| 190 | M | 54 | Left temporal | FIAS, FBTCS | 0 0 | 0 | 6 (39.7) | NA | NA |
| 192 | F | 25 | Multifocal | FIAS | 0 0 | 3 | 4 (30.0) | NA | NA |
| 194 | M | 28 | Right parieto-insular | FAS, FBTCS | 1 1 | 1 | 7 (55.1) | 0.67 | 0.83 |
| 196 | F | 45 | Right frontal | FBTCS, FAS | 0 1 | 1 | 6 (44.8) | NA | -0.20 |
| 198 | F | 19 | Multifocal (bitemporal, left parietal) | FIAS | 3 6 | 18 | 7 (58.9) | 0.17 | 0.10 |
| 199 | F | 29 | Left temporal | FIAS | 1 1 | 1 | 3 (25.0) | 0.00 | 0.00 |
| 201 | F | 30 | Left temporal | FBTCS, FIAS, PNES | 1 1 | 1 | 5 (50.6) | NA | 0.00 |
| 202 | F | 19 | Right temporal | FAS, FBTCS | 0 0 | 4 | 6 (51.6) | -0.30 | -0.20 |
| 205 | M | 26 | Generalized | absences | 0 0 | 0 | 2 (15.1) | NA | NA |
| 206 | M | 40 | Bitemporal | FAS, FIAS | 2 2 | 2 | 8 (62.8) | 0.00 | 0.00 |
| 214 | F | 32 | Left temporal | FAS | 0 0 | 0 | 4 (25.8) | NA | NA |
| 218 | M | 54 | Bitemporal | FIAS | 1 3 | 4 | 7 (39.8) | 0.50 | 0.33 |
| 219 | M | 30 | Multifocal | FIAS, FBTCS | 0 0 | 1 | 4 (28.7) | NA | NA |
| 223 | M | 25 | Left temporal | FAS, FIAS | 0 0 | 0 | 3 (24.7) | NA | NA |
| 224 | F | 19 | Left temporal | FBTCS, FIAS, FAS | 1 2 | 4 | 7 (41.4) | -0.17 | 0.00 |
| 225 | F | 50 | Bitemporal | FIAS | 0 0 | 0 | 6 (49.2) | NA | NA |
| 226 | M | 20 | Right frontal | FIAS | 0 0 | 0 | 5 (36.3) | NA | NA |
| 227 | M | 40 | Right temporal plus | FIAS | 5 5 | 19 | 10 (96.5) | 0.11 | 0.06 |
| 229 | M | 28 | Left temporal plus, right temporal | FBTCS, FIAS | 1 1 | 0 | 3 (20.7) | NA | NA |
| 230 | F | 22 | Right frontal | FBTCS | 0 0 | 0 | 3 (21.3) | NA | NA |
| 231 | M | 39 | Left temporal | FAS | 2 2 | 1 | 8 (69.9) | -0.14 | -0.14 |
| 233 | F | 29 | Bitemporal | FIAS, PNES | 0 0 | 0 | 3 (25.9) | NA | NA |
| 236 | M | 44 | Frontal plus | FIAS, FBTCS | 1 1 | 1 | 12 (93.3) | 0.91 | 0.91 |
| 240 | M | 41 | Left temporal | FAS, FIAS | 1 1 | 0 | 2 (17.6) | NA | NA |
| 241 | M | 40 | Right frontal | FIAS, FBTCS | 1 1 | 1 | 6 (50.9) | 0.20 | 0.40 |
| 242 | M | 33 | Left frontotemporal | FAS, FIAS, FBTCS | 2 5 | 14 | 14 (112.8) | -0.12 | 0.14 |
| 245 | M | 23 | Left temporal plus, right temporal | FBTCS | 0 1 | 1 | 7 (49.9) | NA | 0.67 |
| 246 | M | 51 | Bitemporal | FIAS, FBTCS | 1 1 | 1 | 12 (78.0) | -0.55 | -0.36 |

*Most epilepsy foci were not validated by intracranial EEG investigations.

**Abbreviations**

Main seizure type

FAS: Focal aware seizure

FIAS: Focal impaired awareness seizure

FBTCS: Focal to bilateral to tonic-clonic seizure

GTCS: Generalized tonic-clonic seizure

PNES: Psychogenic nonepileptic seizure
